# Adie’s pupil and systemic manifestations: a rare unilateral presentation

**DOI:** 10.3205/oc000246

**Published:** 2024-12-03

**Authors:** M N Vimisha, M Shah Virna, Kumar Karthik, R Sharanya

**Affiliations:** 1 Department of Neuro-Ophthalmology, Aravind Eye Hospital and Postgraduate Institute, Coimbatore, India

## Abstract

We present a case of a young woman who presented with blurring of vision in her right eye, worsening on near work. Detailed ophthalmic and neurological evaluation was done, which revealed light near dissociation, vermiform iris movements, constriction to diluted pilocarpine with absent deep tendon reflexes. Laboratory investigation indicated mild iron deficiency anemia and reduced vitamin D3 level. On orthopedic evaluation she was diagnosed with right knee joint arthritis and grade 1 patellar chondromalacia. Neuroimaging was within normal limits. Magnetic resonance imaging (MRI) pelvis and lumbar spine showed left ovarian cyst, small periurethral cyst and small hemagioma in left sacral ala. A diagnosis of Holmes-Adie syndrome was made and she was prescribed photochromatic glasses.

## Introduction

Holmes-Adie syndrome was described in 1932, with a present incidence of 4.7/100,000 and prevalence of 2/1,000 cases per year, more commonly in females (2.6:1) in their third decade and 80% unilateral [1]. It is mostly idiopathic or results from parasympathetic denervation at the level of the ciliary ganglion or short ciliary nerves characterized by light near dissociation, segmental iris constriction, vermiform movements of the pupillary border, and hypersensitivity to 0.125% pilocarpine. It is associated with absent deep tendon reflexes and may also present with autonomic dysfunction such as cough, orthostatic hypotension, impaired cardiovascular reflexes, diarrhea, hyperhidrosis or hypohydrosis (Ross or Harlequin syndromes). Miotics like pilocarpine and reading glasses for near vision can be prescribed to reduce symptoms. 

## Case description

**Figure 1:**
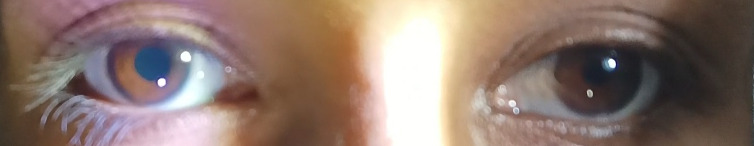
The right pupil shows a sluggish reaction to light.

**Figure 2:**
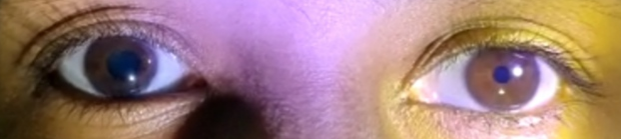
The left pupil shows a brisk reaction to light.

**Figure 3:**
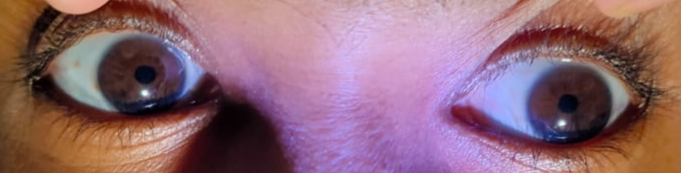
Both pupils respond normally to near targets.

A 34-year-old woman presented to our neuro-ophthalmology department with blurred vision in her right eye, particularly in the right temporal field, over the past week, which worsened with near work. She reported no additional ophthalmological or systemic symptoms and denied trauma, diplopia, ptosis, use of eye drops, or headaches. Her medical history and systemic health were unremarkable. Best corrected visual acuity was 6/6 in both eyes, and color vision was normal. Examination revealed normal afferent visual function, no ptosis, and full extraocular movements. However, she had anisocoria with the right pupil larger (5 mm) compared to the left (4 mm) in dim light, and the difference was more pronounced in bright light (torch light) (5 mm in the right eye and 2 mm in the left), suggesting an abnormal right pupil. The right pupil was sluggish in reaction to light (Figure 1 (Fig. 1)) with vermiform movement observed on slit lamp examination, while the left pupil was briskly reactive (Figure 2 (Fig. 2)). Both pupils responded normally to near targets (Figure 3 (Fig. 3)). The anterior and posterior segment examinations were normal. Neurological evaluation showed absent knee and ankle jerks on the right side. Pilocarpine testing showed a significant reduction in the size of the right pupil with 0.125% pilocarpine. Laboratory tests indicated mild iron deficiency anemia with reduced hemoglobin, packed cell volume, mean corpuscular volume, and mean corpuscular hemoglobin, and increased red cell distribution width. Serum calcium levels were normal, but vitamin D3 was low. Magnetic resonance imaging of the brain and orbit showed no abnormalities. An orthopedic consultation diagnosed early arthritis/reactive arthritis and grade 1 patella chondromalacia of the right knee. Magnetic resonance imaging of the pelvis and lumbar spine revealed a left ovarian cyst, small periurethral cyst, and a small hemangioma in the left sacral ala, none of which required treatment. Pilocarpine eye drops (0.125%) were prescribed for the right eye but discontinued after two weeks due to lack of improvement. The patient was given photochromatic glasses and reassured about the benign nature of her condition. She remains under close follow-up, as no similar systemic associations have been reported in other cases of Adie syndrome.

## Discussion

Conditions that could mimic or cause Adie syndrome, including systemic dysautonomia, syphilis, diabetes, chronic alcoholism, encephalitis, multiple sclerosis, peripheral nerve disease, paraneoplastic syndromes, midbrain tumors, herpes zoster, neurosarcoidosis, ischemic disease, trauma or mechanical compression which can damage the ciliary ganglion, were excluded in our patient. Adie syndrome does not have a progressive course or causes any morbidity or mortality. The loss of deep tendon reflexes is permanent and may progress over time. Most patients require only reassurance after confirmation of the diagnosis. Topical low-dose pilocarpine or physostigmine drops may be administered as a treatment. Full-strength pilocarpine 1%–4% has significant ocular side effects such as intraocular inflammation, periocular pain and peripheral retinal tears, which is why the dilute formulation is favoured [2]. For those failing conservative management with drug therapy, thoracic sympathectomy is the treatment of choice for diaphoresis [3].

There have been reports of rare associations of angle-closure glaucoma with Adie pupil [4], case reports of Holmes-Adie syndrome as an early manifestation of systemic lupus erythematosus [5], bilateral tonic pupils as the initial manifestation of Sjögren’s Syndrome [6], association between Vogt Koyanagi Harada disease and tonic pupil, the tonic pupil persisting after other clinical features of this syndrome had disappeared [7], post covid infection [8], associations with idiopathic sclerochoroidal calcification [9], neurosyphilis [10], and a case of Adie’s tonic pupil resolved after treatment of ovarian endometriosis [11]. There have been studies showing association of rheumatoid arthritis and endometriosis with inflammation and autonomic dysfunction [12], polycystic ovarian syndrome with autonomic dysfunction [13] and autonomic dysfunction preceding the development of rheumatoid arthritis [14]. Rarely, tonic pupil might occur in giant cell arteritis in elderly patients; our patient was relatively young and did not have any symptoms suggestive of giant cell arteritis. However, these unusual systemic findings in our patient may be coincidental. We are keeping a close follow-up to monitor for any additional rare associations.

Usually, an improvement in accommodation is achieved by spontaneous partial regeneration of the damaged neurons within a few months of onset of the disease [15]. The pupil light reaction becomes weaker over time with an increasing light-near dissociation, and the pupil becomes smaller with time (“little old Adie”).

## Conclusion

To conclude, Holmes-Adie syndrome is a rare condition encountered in clinical settings. Unilateral tonic pupil, especially without accompanying symptoms suggesting autonomic dysfunction, inflammation, or systemic effects, is typically idiopathic and benign. However, the presence of additional signs necessitates thorough assessment to distinguish between Holmes-Adie syndrome, Ross syndrome, Harlequin syndrome, oculomotor nerve palsy, anticholinergic drug use, and congenital mydriasis. Effective management involves coordinated efforts among neurologists and ophthalmologists to ensure early detection and optimize patient care.

## Notes

### Competing interests

The authors declare that they have no competing interests.
